# Morphometrics and stable isotopes differentiate wintering populations of a migratory bird

**DOI:** 10.1186/s40462-016-0085-6

**Published:** 2016-08-02

**Authors:** Ivan Maggini, Benjamin Metzger, Maren Voss, Christian C. Voigt, Franz Bairlein

**Affiliations:** 1 Institute of Avian Research “Vogelwarte Helgoland”, Wilhelmshaven, Germany; 2 Konrad-Lorenz Institute of Ethology, University of Veterinary Medicine, Vienna, Austria; 3 BirdLife Malta, Xemxija, Malta; 4 Leibniz-Institute of Baltic Sea Research Warnemünde, IOW, Rostock, Germany; 5 Leibniz Institute for Zoo and Wildlife Research, Berlin, Germany

**Keywords:** Sahel, East Africa, Wintering, *Oenanthe oenanthe*, Connectivity

## Abstract

**Background:**

Describing migratory connectivity in mobile animals is crucial for understanding the selective pressures acting on different populations throughout their life cycle. Tracking single individuals has provided valuable data, but for most species the data available are still spurious and usually limited to a few individuals. Since different populations of migratory birds can be distinguished by a combination of morphometric measurements and the isotopic composition of their feathers, it is possible to measure these parameters on a large sample to differentiate populations.

**Methods:**

We studied northern wheatears, *Oenanthe oenanthe*, captured in their African wintering range and applied discriminant analyses on morphometric measurements and stable isotope signatures to determine whether birds found in different areas were distinguishable from each other.

**Results:**

Morphometric and isotopic measurements alone were not sufficient to discriminate between the birds of ssp. *oenanthe* from different areas in Africa. When combining the two measurements, however, assignment to the different groups became substantially more accurate. Following the discriminant analysis of morphometrics and δ^2^H, δ^13^C, and δ^15^N isotopes signatures, 19 of 20 *oenanthe* from Kenya, 15 of 20 *oenanthe* from Mali/Mauritania, and 19 of 20 *oenanthe* from Niger were assigned correctly to their wintering area.

**Conclusions:**

Our results show that birds at different wintering sites can be distinguished from each other when using a combination of markers. We discuss the possible breeding origins of these wintering birds.

**Electronic supplementary material:**

The online version of this article (doi:10.1186/s40462-016-0085-6) contains supplementary material, which is available to authorized users.

## Background

In migratory animals, reproductive success and survival are not only affected by conditions found during the breeding season, but are influenced by conditions experienced during the non-breeding period as well [1–10]. In the case where the non-breeding range is completely disjoint from the breeding areas, such as for long-distance migratory birds, environmental factors encountered during the non-breeding period can affect factors such as pre-migratory fueling and migratory departure timing [11–15]. In addition, selective pressures and mortality rates vary between the breeding, non-breeding and migratory seasons [16, 17]. These aspects can ultimately carry over into reproductive success, which in many cases is affected by arrival timing and body condition at arrival [15, 18–28]. In order to understand the mechanisms that contribute to carry-over effects and eventually population dynamics, it is therefore important to establish the connectivity between the breeding and non-breeding range of different populations of a given species.

In recent times, technological advances have increased our knowledge about migratory connectivity in several species. The use of satellite telemetry allowed detailed description of the migratory tracks of medium to large-sized birds (e.g. [29–31]), while light-level geolocators have been also used in smaller species (e.g. [32–35]). These methods have added conspicuously to the existing knowledge that was mostly based on, sometimes scant, recoveries of marked animals. However, there are drawbacks to the use of such methods, such as the possible effects of tracking devices on the behaviour of the individuals, and the small sample sizes.

A different method that can be used to link birds at a particular non-breeding site with their breeding population is to take advantage of morphological or biochemical markers that distinguish birds breeding at different sites. In many species of migratory birds, it is known that populations can be differentiated by morphological features, such as differences in plumage or size [36–38]. In particular, it is well known that wing morphology is strictly correlated with migratory distance, with birds migrating further having longer and more pointed wings than birds migrating shorter distances [39–43]. In addition, the chemical properties of feathers, and in particular their stable isotope composition, can give helpful insights into the location where the feathers were grown [11, 44–50]. Considering these differences, it is possible to differentiate birds in their non-breeding range and assign them to their breeding population.

The northern wheatear (*Oenanthe oenanthe*) is a long-distance migrant breeding in the Holarctic region and wintering in sub-Saharan Africa [51]. It is one of the migratory Passerine birds with the most extended distribution range. Population trends in Europe show differing patterns, with increases in some populations and decreases in others [52]. These differences may be caused by varying survival rates due to the use of different wintering areas. Since we know that migratory traits in this species are endogenously determined and population-specific [53, 54], we would expect a relatively strict differentiation in the location of wintering areas among populations. The use of geolocators revealed the possibility of strict population-specific migratory orientation but wintering sites were relatively scattered across large areas [33, 34, 55]. However, for the moment our knowledge is limited to a few populations, and comes from just a few individuals.

In this study, we captured northern wheatears in their non-breeding range in Africa before the beginning of their northward migration in spring. We collected morphometric data and measured stable isotope composition of feathers that were grown in the breeding areas in order to assign each individual to a group. Northern wheatears occur in three morphologically distinct subspecies: the long-winged and darker coloured *leucorhoa* from Iceland, Greenland and Northeast Canada; *seebohmi* from the Atlas mountain range in North Africa which has a black throat and black underwing coverts; and *oenanthe* occurring in the whole rest of the range, from Europe through Siberia to western North America (Alaska). While the first two subspecies are readily identifiable when captured, different populations of the *oenanthe* subspecies cannot be easily separated. However, we know that it is possible to assign birds to their breeding population with good probability when combining morphometric measurements and stable isotope signatures of their feathers [56, 57]. Therefore, we predicted that if migratory connectivity is high, birds captured at different sites in the wintering range would differ from each other. We took samples from birds captured at several sites in sub-Saharan Africa that we divided in three main regions: the westernmost area located in southern Mauritania/northwest Mali (hereafter Mauritania/Mali), the western-central area in Niger, and the eastern area in Kenya. We expected to find differences in morphometric measurements and stable isotope compositions of feathers among birds from these three main areas. Given the lack of reference material from the breeding grounds, our project focused on the differentiation of distinct wintering populations.

## Methods

**Fig. 1:**
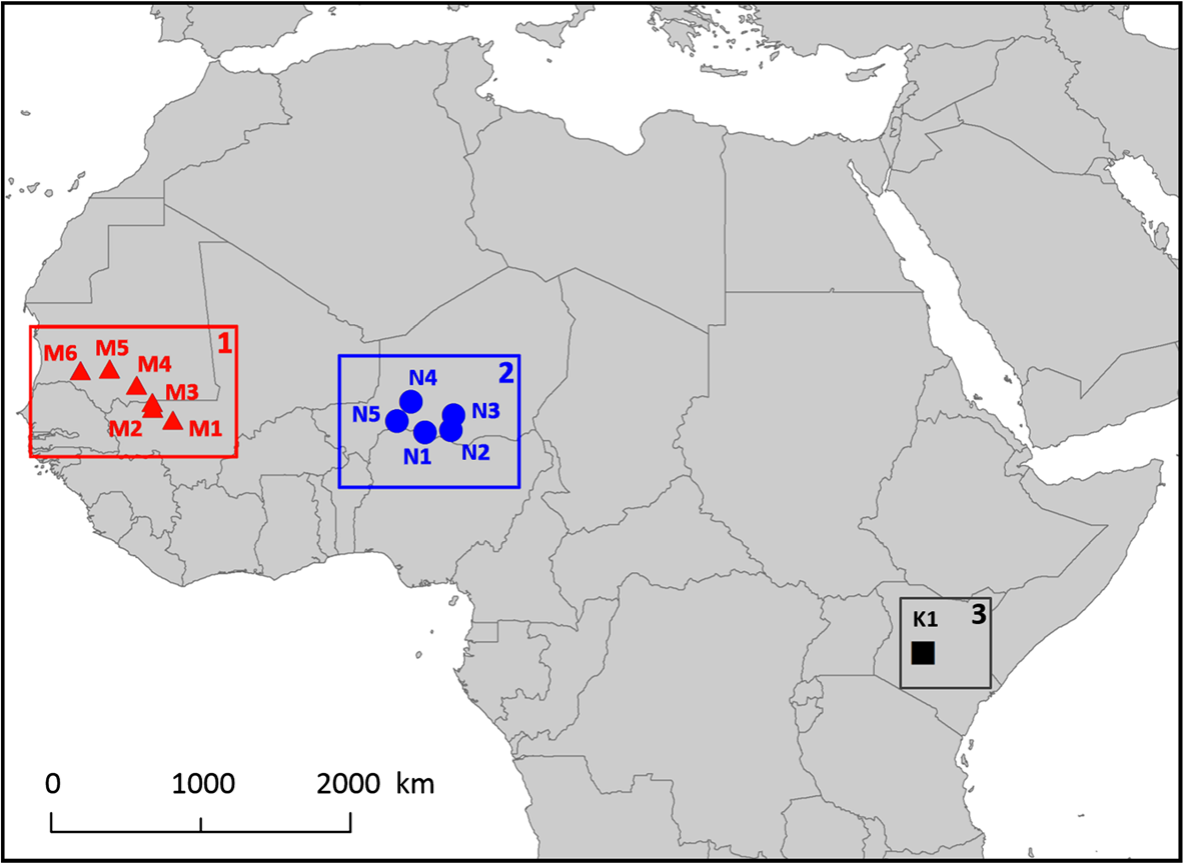
Map of the study sites in sub-Saharan Africa. Area 1 represents Mauritania/Mali, area 2 represents Niger and area 3 represents Kenya. M1: Boucle de Baoulé (14.31°N; 8.18°W); M2: 30 km S of Nioro (15.03°N; 9.37°W); M3: 20 km N of Nioro (15.37°N; 9.43°W); M4: 80 km W of Ayoun (16.43°N; 10.36°W); M5: Massif de Bellar (17.38°N; 11.98°W); M6: 30 km E of Aleg (17.29°N; 13.71°W); N1: Tibiri (13.58°N; 7.04°E); N2: Takieta (13.69°N; 8.58°E); N3: Tanout (14.61°N; 8.75°E); N4: Abalak (15.42°N; 6.19°E); N5: Awillikiss (14.24°N; 5.36°E); K1: Mpala Research Centre (0.33°N; 36.90°E)

Northern wheatears were captured at 6 different sites in Mauritania/Mali and 5 different sites in Niger (Fig. 1) during February-March 2007 and at one site in Kenya (Fig. 1) in January-February 2008. See Fig. 1 for a full description of the sites and their coordinates. Usually, wheatears were found in the vicinity of human settlements in open areas with cultivations.

Wheatears were captured with spring traps baited with mealworms during the morning hours. Upon capture, the birds were assigned to their subspecies, sexed and aged following [58]. We assigned birds to ssp. *leucorhoa* when their plumage was darker and the length of the wing chord was >99 mm in males and >96 mm in females [59]. We assigned birds to ssp. *seebohmi* when they had completely black underwing coverts. In addition, males of this subspecies have a dark throat. All other birds were assigned to ssp. *oenanthe*. We then took a set of measures from all captured birds: tarsus, wing chord, the length of the 8 outermost primaries (P9-P2), bill length (measured between the tip of the bill and the skull), bill depth and bill width measured at the nostrils following standard protocols [60]. After measuring, we sampled the second outermost tail feather for isotopic analysis. The birds were banded and released within 20 min from capture. From the length of the 8 primary feathers we calculated indices of wing pointedness and concaveness using formulas from [41]. In the following text we will refer to Lockwood’s c2 index for pointedness and c3 index for concaveness.

The δ^13^C and δ^15^N values were measured at the Leibniz-Institute for Baltic Sea Research (Rostock, Germany). The samples were cut from the tip of one feather per bird, 0.50–0.75 mg weighed into tin capsules. Analysis of the isotope ratios was performed by a CN-Analyzer (model 1108, CE Instruments, Thermo) connected online to an isotope ratio mass spectrometer (IRMS) (Delta S, Thermo-Finnigan, Bremen) via an open split interface. Reference gases came from ultra-pure N_2_ and CO_2_ cylinders calibrated using IAEA standards. For nitrogen routine standard gas calibration was done with N1 (δ^15^N 0.4 ‰), N2 ((δ^15^N -20.3 ‰), and N3 ((δ^15^N 4.7 ‰), while the CO_2_ gas was calibrated with NBS 22 (δ^13^C -29.7 ‰) and IAEA-CH6 (δ^13^C -10.8 ‰). Values are reported relative to atmospheric N_2_ (δ^15^N) and VPDB (δ^13^C- Vienna Peedee belemnite). Wassenaar [61] gives further information about the mechanism of the analysis and the composition of the standards. The precision of the measurements was better than 0.2 ‰ as determined from an internal reference substance (peptone, Merck) interspersed after each fifth sample. The δ^2^H values were measured at the stable isotope laboratory of the Leibniz Institute for Zoo and Wildlife Research, Berlin, Germany, using an isotope ratio mass spectrometer (Delta V Advantage; Thermo Finnigan, Bremen, Germany) connected to an elemental analyser (HT Elementaranalysator; HEKAtech, Wegberg, Germany) (see [62] for details). We used the international standards IAEA NBS 22 (δ^13^C -30.3 ‰) (mineral oil) and IAEA-CH-7 (polyethylene) (δ^13^C -32.15 ‰) to determine the stable isotope ratio of the reference gas used in the mass spectrometer. We clipped 350 ± 7 μg of feather tissue from the tip of the feather and loaded it in silver capsules (IVA Analysetechnik, Meerbusch, Germany). These were kept in a microtiter tray over 1 week to equilibrate with ambient air. Subsequently, trays were stored for at least 24 h at 50 °C in a drying oven. We used the comparative equilibration method [63] to account for the amount of exchangeable hydrogen in feather keratin. We used three standards that covered the range of expected δ^2^H values in our samples and to determine the δ^2^H of non-exchangeable hydrogen [63, 64]. The stable hydrogen isotope ratios of the non-exchangeable hydrogen (mean δ^2^H_n_ ± 1SD) of the standards were: −133.6 ± 1.2 ‰, −109.1 ± 1.2 ‰, and −87.2 ± 1.0 ‰. In the sequential order of one autorun, keratin standards were placed at positions 1–6 (2 × 3 standards) and at any 9–11th position (three standards each time). Analytical precision based on the repeated analyses of laboratory keratin standards was always better than 0.9 ‰ (1SD). Isotope ratios are given in the usual delta notation as the deviation from a standard substance.

The statistical analysis was performed only on birds from ssp. *oenanthe*. The birds were divided into three main geographic groups according to the location of capture (Mauritania/Mali, Niger, and Kenya). First, we used two-way ANOVA to determine whether there were differences among sexes and sites and the sex*site interaction in morphometric (wing, tarsus, and bill length, bill width and depth, c2 and c3-indexes) and isotope measurements (δ^2^H, δ^13^C, and δ^15^N). We then performed separated analyses for each sex using one-way ANOVAs for the same variables. If significant effects were found, we performed pairwise comparisons using Bonferroni corrections. In the last step of the analysis we performed a linear discriminant analysis. Since we had three main groups, the analysis generated two linear discriminator functions (LD1 and LD2). These were used to predict with which probability an individual was assigned to one of the three areas. We then evaluated how many cases were assigned to the correct area, considering only individuals for which the predicted likelihood was >70 %. If the likelihood was under 70 %, we considered the assignment to be unknown. First, we performed this analysis using only morphometric measurements (tarsus, wing chord, bill length, bill width, bill depth, c2 and c3 indices), and then including the three stable isotope signatures. Since we only measured δ^2^H in 20 individuals per area, we had a reduced sample size for this analysis. Wilks’ lambda was calculated to test whether there were differences between the group’s means on the combination of dependent variables used for discriminant analysis. All analyses were performed separately for males and females. The use of parametric models was justified since all variables were normally distributed, as determined by Shapiro-Wilk tests (*p* > 0.05 for all tests), and variances were homogeneous as determined by Levene’s test (*p* > 0.05 for all tests). The assumptions for using linear discriminant analysis were all met (normality, homogeneous variances, and correlation among variables, as shown by variance inflation factors always <3). We used software R 3.0.2 for the analysis [65].

## Results

We captured 107 northern wheatears in this study. Six birds belonged to ssp. *leucorhoa*, 9 to ssp. *seebohmi*, and 92 to ssp. *oenanthe. leucorhoa* and *seebohmi* were only found in Mauritania/Mali, while *oenanthe* were found in all three areas (Mauritania/Mali: 27; Niger: 37; Kenya: 28 individuals). Wing length (two-way ANOVA. Site: *p* = 0.013, Sex: *p* < 0.001, Site*Sex: *p* = 0.877), tarsus length (Site: *p* = 0.002, Sex: *p* < 0.001, Site*Sex: *p* = 0.233), bill length (Site: *p* = 0.004, Sex: *p* < 0.001, Site*Sex: *p* = 0.060), and c2-index (Site: *p* = 0.032, Sex: *p* = 0.012, Site*Sex: *p* = 0.780) differed between sites and sexes. There were no differences between site and sex in bill width (Site: *p* = 0.289, Sex: *p* = 0.813, Site*Sex: *p* = 0.086), bill depth (Site: *p* = 0.239, Sex: *p* = 0.127, Site*Sex: *p* = 0.932), and c3-index (Site: *p* = 0.750, Sex: *p* = 0.584, Site*Sex: *p* = 0.918). δ^2^H differed significantly among sites, and there were site-specific differences between sexes (Site: *p* < 0.001, Sex: *p* = 0.196, Site*Sex: *p* = 0.035). A visual inspection of the plots (not represented here) showed that δ^2^H values were lower in males than in females in Niger. Differences among sites but not among sexes were found for δ^15^N (Site: *p* < 0.001, Sex: *p* = 0.798, Site*Sex: *p* = 0.564) and δ^13^C (Site: *p* = 0.001, Sex: *p* = 0.144, Site*Sex: *p* = 0.227).

### Males

Table 1 shows the average values for morphometric measurements and isotope signatures in *oenanthe* males from the different sites. Wing length differed significantly among groups (one-way ANOVA: F_2,55_ = 26.896, *p* = 0.033). It was highest in males from Kenya which differed significantly from Niger (*p* = 0.045) but not from Mauritania/Mali (*p* = 0.085). Males from Niger did not differ from Mauritania/Mali in wing length (*p* > 0.5).

**Table 1:** Morphometric measurements and stable isotope values for males of the ssp. *oenanthe* in three different regions of their wintering range. Values are given ± SD

	Kenya (*N* = 14) ^a^	Mauritania/Mali (*N* = 16) ^a^	Niger (*N* = 29) ^a^
Wing chord [mm]	99.3 ± 2.4	97.7 ± 4.4	97.0 ± 2.4
Tarsus [mm]	28.4 ± 0.9	27.4 ± 0.8	27.2 ± 1.0
Bill length [mm]	20.5 ± 0.6	20.0 ± 0.8	19.5 ± 0.7
Bill depth [mm]	4.1 ± 0.2	4.2 ± 0.3	4.0 ± 0.3
Bill width [mm]	3.8 ± 0.2	3.8 ± 0.2	3.7 ± 0.2
c2-index	0.36 ± 0.09	0.28 ± 0.11	0.29 ± 0.13
c3-index	−1.08 ± 0.13	−1.13 ± 0.19	−1.12 ± 0.19
δ^2^H [‰] ^a^	−47.39 ± 5.56	−52.94 ± 12.20	−65.29 ± 8.96
δ^15^N [‰]	7.79 ± 1.47	6.76 ± 2.36	10.85 ± 2.48
δ^13^C [‰]	−23.23 ± 0.70	−23.39 ± 0.75	−22.58 ± 1.16

^a^ Sample sizes for δ^2^H. Kenya: *N* = 11; Mauritania/Mali: *N* = 13; Niger: *N* = 14

There were significant differences in tarsus length among groups (F_2,55_ = 7.552, *p* = 0.001). Males from Kenya had longer tarsi than males from Mauritania/Mali (*p* = 0.010) and from Niger (*p* < 0.001). Males from Niger did not differ from Mauritania/Mali in tarsus length (*p* > 0.5).

Bill length differed significantly among groups (F_2,55_ = 13.88, *p* < 0.001). Males from Niger had the shortest bills. The difference was significant from males from Kenya (*p* < 0.001) and from Mauritania/Mali (*p* = 0.050). Males from Mauritania/Mali did not differ significantly in bill length from Kenya (*p* = 0.052). Bill width did not differ among groups (F_2,55_ = 2.060, *p* = 0.137), nor did bill depth (F_2,55_ = 0.887, *p* = 0.418).

No differences in wing pointedness (c2-index) were found among groups (F_2,55_ = 1.811, *p* = 0.173). Similarly, wing concaveness (c3-index) did not differ among groups (F_2,55_ = 0.301, *p* = 0.742).

δ^2^H values differed significantly among groups (F_2,37_ = 12.61, *p* < 0.001). *oenanthe* males from Niger had the lowest δ^2^H values and differed significantly from Kenya (*p* < 0.001) and Mauritania/Mali (*p* = 0.005). Birds from Kenya and Mauritania/Mali did not differ from each other in δ^2^H (*p* = 0.428).

δ^15^N values differed significantly among groups (F_2,55_ = 18.74, *p* < 0.001). The δ^15^N values were highest in males from Niger and differed significantly from both Kenya and Mauritania/Mali (*p* < 0.001 in both pairwise comparisons). Males from Kenya and Mauritania/Mali did not differ from each other in δ^15^N (*p* > 0.5).

δ^13^C values showed a significant difference among groups (F_2,55_ = 4.571, *p* = 0.015). Birds from Mauritania/Mali had the lowest δ^13^C values and differed significantly from Niger (*p* = 0.023) but not from Kenya (*p* > 0.5). Males from Kenya did not differ significantly from Niger in δ^13^C (*p* = 0.133).

**Fig. 2:**
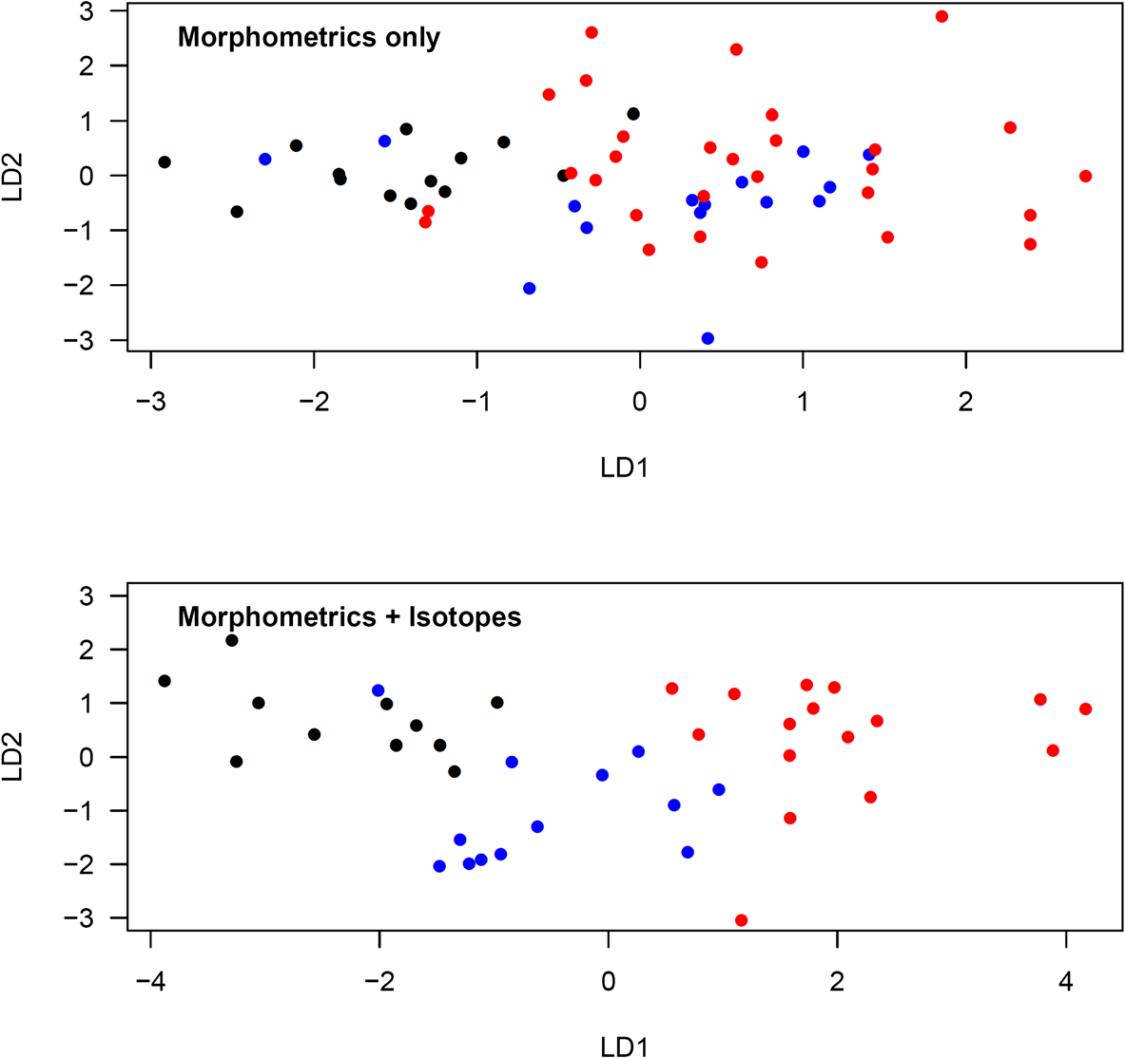
Discriminant analysis ordination for *oenanthe* males from different study sites using morphometric measurements only or morphometric and isotopic measurements combined. Morphometrics only: LD1 = −0.54*Tarsus-0.08*Wing chord-0.84*Bill length-0.50*Bill width + 0.37*Bill depth-4.50*c2-0.53*c3; LD2 = −0.13*Tarsus + 0.22*Wing chord-0.41*Bill length-2.40*Bill width-1.86*Bill depth + 5.13*c2-0.27*c3. Morphmetrics and isotopes: LD1 = −0.26*Tarsus-0.27*Wing chord-1.03*Bill length-0.35*Bill width-0.91*Bill depth-1.09*c2-1.21*c3-0.09*δ^2^H + 0.26*δ^15^N + 0.02*δ^13^C; LD2 = 0.36*Tarsus + 0.13*Wing chord + 0.66*Bill length-0.80*Bill width-0.67*Bill depth + 5.97*c2 + 0.06*c3-0.04*δ^2^H + 0.25*δ^15^N + 0.15*δ^13^C. Red: Mauritania/Mali, blue: Niger, black: Kenya

Figure 2 shows the discriminant ordinations for the analysis of morphometric measurements only, and of morphometric measurements and isotopes combined, in *oenanthe* males. When considering morphometric measurements alone, the proportions of trace were 88.5 % for LD1 and 11.5 % for LD2. According to these functions, 6 of 14 (42.9 %) *oenanthe* males captured in Kenya were correctly assigned (8 unknown). Only one of 15 (6.7 %) *oenanthe* males captured in Mauritania/Mali was correctly assigned (9 unknown, 2 incorrectly assigned to Kenya and 3 incorrectly assigned to Niger). 13 of 29 (44.8 %) *oenanthe* males captured in Niger were correctly assigned (16 unknown). Wilks’ lambda was 0.518 (df = 2,55; *p* = 0.002). When considering both morphometric and isotopic measurements, the proportions of trace were 87.7 % for LD1 and 12.3 % for LD2. According to these functions, all 11 (100 %) *oenanthe* males captured in Kenya were correctly assigned. Ten out of 13 (76.9 %) *oenanthe* males captured in Mauritania/Mali were correctly assigned (2 unknown, one incorrectly assigned to Kenya). Fifteen out of 16 (93.8 %) *oenanthe* males captured in Niger were correctly assigned (one incorrectly assigned to Mauritania/Mali). Wilks’ lambda was 0.146 (df = 2,37; *p* < 0.001).

### Females

Table 2 shows the average values for morphometric measurements and isotope signatures in females of the different groups. There were no significant differences among groups in female wing length (F_2,31_ = 2.975, *p* = 0.066), tarsus length (F_2,31_ = 2.425, *p* = 0.105), bill length (F_2,31_ = 0.132, *p* = 0.877), bill width (F_2,31_ = 1.840, *p* = 0.176), and bill depth (F_2,31_ = 1.014, *p* = 0.374). Neither wing pointedness (c2-index) nor wing concaveness (c3-index) differed significantly among groups (c2-index: F_2,31_ = 0.617, *p* = 0.546; c3-index: F_2,31_ = 0.075, *p* = 0.928).

**Table 2:** Morphometric measurements and stable isotope values for females of the ssp. *oenanthe* in three different regions of their wintering range. Values are given ± SD

	Kenya (*N* = 14) ^a^	Mauritania/Mali (*N* = 11) ^a^	Niger (*N* = 8) ^a^
Wing chord [mm]	95.8 ± 3.1	93.2 ± 2.4	94.0 ± 2.9
Tarsus [mm]	27.0 ± 0.6	26.5 ± 0.9	26.6 ± 0.6
Bill length [mm]	19.8 ± 0.5	19.8 ± 0.9	19.5 ± 0.5
Bill depth [mm]	4.0 ± 0.2	4.1 ± 0.2	4.0 ± 0.3
Bill width [mm]	3.7 ± 0.2	3.8 ± 0.2	3.8 ± 0.2
c2-index	0.42 ± 0.13	0.36 ± 0.19	0.34 ± 0.09
c3-index	−1.12 ± 0.16	−1.14 ± 0.12	−1.14 ± 0.10
δ^2^H [‰] ^a^	−49.36 ± 6.34	−53.78 ± 5.60	−50.88 ± 13.37
δ^15^N [‰]	7.69 ± 1.74	7.39 ± 2.53	9.98 ± 3.40
δ^13^C [‰]	−23.20 ± 0.56	−22.66 ± 1.25	−21.48 ± 2.52

^a^ Sample sizes for δ^2^H. Kenya: *N* = 9; Mauritania/Mali: *N* = 7; Niger: *N* = 4

Neither δ^2^H nor δ^15^N values differed significantly among groups (δ^2^H: F_2,17_ = 0.631, *p* = 0.544; δ^15^N: F_2,31_ = 3.186, *p* = 0.055). Only δ^13^C values differed significantly among groups (F_2,31_ = 3.616, *p* = 0.039). Females from Niger had the highest values, which were significantly different from Kenya (*p* = 0.034) but not from Mauritania/Mali (*p* = 0.271). Females from Kenya and Mauritania/Mali did not differ significantly in δ^13^C (*p* > 0.5).

**Fig. 3:**
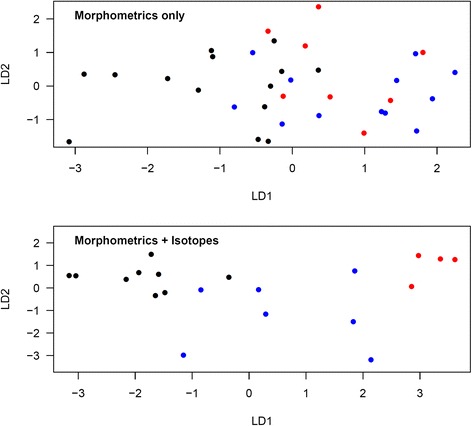
Discriminant analysis ordination for *oenanthe* females from different study sites using morphometric measurements only or morphometric and isotopic measurements combined. Morphometrics only: LD1 = −0.53*Tarsus-0.32*Wing chord-0.41*Bill length + 2.36*Bill width + 2.79*Bill depth-4.22*c2-0.57*c3; LD2 = 0.92*Tarsus-0.01*Wing chord-1.30*Bill length + 3.15*Bill width-2.78*Bill depth-2.36*c2 + 0.97*c3. Morphometrics and isotopes: LD1 = −2.68*Tarsus-0.01*Wing chord + 0.89*Bill length-1.31*Bill width + 1.97*Bill depth-5.63*c2-6.80*c3-0.10*δ^2^H + 0.56*δ^15^N + 0.00*δ^13^C; LD2 = 0.02*Tarsus + 0.09*Wing chord-0.89*Bill length-0.99*Bill width + 0.36*Bill depth + 2.90*c2-0.39*c3 + 0.04*δ^2^H + 0.54*δ^15^N-1.02*δ^13^C. Red: Mauritania/Mali, blue: Niger, black: Kenya

Figure 3 shows the discriminant ordinations for the analysis of morphometric measurements only, and of morphometric measurements and isotopes combined, in *oenanthe* females. When considering morphometric measurements alone, the proportions of trace were 86.4 % for LD1 and 13.6 % for LD2. According to these functions, 7 of 14 (50.0 %) *oenanthe* females captured in Kenya were correctly assigned (7 unknown). 4 of 12 (33.3 %) *oenanthe* females captured in Mauritania/Mali were correctly assigned (7 unknown, one incorrectly assigned to Kenya). One of 8 (12.5 %) *oenanthe* females captured in Niger were correctly assigned (5 unknown, 2 incorrectly assigned to Mauritania/Mali). Wilks’ lambda was 0.482 (df = 2,31; *p* = 0.120). When considering both morphometric and isotopic measurements, the proportions of trace were 82.8 % for LD1 and 17.2 % for LD2. According to these functions, 8 out of 9 (88.9 %) *oenanthe* females captured in Kenya were correctly assigned (one incorrectly assigned to Mauritania/Mali). Five out of 7 (71.4 %) *oenanthe* females captured in Mauritania/Mali were correctly assigned (one incorrectly assigned to Kenya, one incorrectly assigned to Niger). Four out of 4 (100 %) *oenanthe* females captured in Niger were correctly assigned. Wilks’ lambda was 0.095 (df = 2,17; *p* = 0.119).

## Discussion and conclusions

We found birds of all three wheatear subspecies in their wintering grounds in sub-Saharan Africa. The morphologically distinct *leucorhoa* and *seebohmi*, readily identifiable in the field, were found only in the westernmost section of the area, namely southern Mauritania and NW-Mali (see also [66, 67] for *seebohmi*). The observed wintering distribution of *leucorhoa* confirmed the information obtained from a bird of this subspecies fitted with a geolocator in Eastern Canada [33]. The *oenanthe* subspecies was present in the whole study area. The analysis of morphological traits and stable hydrogen, carbon, and nitrogen isotopes gave additional insights into the differentiation among its wintering populations. Our results show that a combination of multiple markers provides an acceptable resolution to differentiate birds at their wintering grounds.

Since the breeding range of the northern wheatear is extremely broad [51] it is challenging to collect reference material from the breeding areas in order to compare it with samples collected in their non-breeding range. However, it is possible to refine our assignments by critically analyzing the information collected. Wheatears of ssp. *oenanthe* sampled in different areas were quite similar in terms of morphometric measurements. Only males found in Kenya were on average larger than males from Mauritania/Mali and Niger. Wing length and pointedness have been shown to be connected with migration distance in several species [39–42] including the wheatear [43]. However, we found no significant differences in the wheatears from our sample. This observation underlines the poor performance of morphometric measurements alone for identifying wintering populations of the wheatear. In particular, females did not show any morphological differences across the wintering area.

Stable isotope ratios of feathers have increasingly been used to refine assignments of birds to their breeding population [11, 44–49]. δ^2^H values show latitudinal and altitudinal trends depending on precipitation [68] with birds from higher latitudes and elevations showing the lowest ratios. δ^13^C values depend mostly on the plant photosynthetic pathways (and on the proportion of C4 relative to C3 plants, the latter having lower δ^13^C because of their different photosynthetic pathways and enzymes used) [46, 69–71]. δ^15^N values are related to the distance from the sea [72] or to dietary stress and anthropogenic influx from e.g. agriculture [71, 73–75], with higher values further away from the coast and with higher anthropogenic influx. Both δ^13^C and δ^15^N values decrease with increasing annual rainfall [76]. In this study, we found differences in δ^13^C values in both males and females. Birds from Niger showed higher values than birds from both other regions, indicating that wheatears probably originated from different vegetation zones, or that they feed in differently vegetated habitats within their breeding range. Higher proportions of C4 plants in Europe are found in the Mediterranean region and in the steppes of Eastern Europe [77], suggesting that birds in Niger might originate from these areas. In addition, δ^15^N values were highest in *oenanthe* males from Niger, which also had the lowest δ^2^H values. This suggests that *oenanthe* males wintering in Niger might originate from inland areas with low annual rainfall. This difference was not found in females, suggesting that female distribution in winter might overlap more widely than the male’s. Interestingly, birds from the two most distant areas in our study (Mauritania/Mali and Kenya) did not differ in either δ^2^H nor δ^15^N. This similarity is unlikely to be due to similar breeding origins, but might rather be related to similar habitats in distant breeding areas. In general, all δ^2^H values measured in our study were relatively high when compared to those measured at different sites of Northern Europe [56], suggesting that we might have missed birds breeding the furthest North, such as Scandinavian or Scottish birds.

According to geolocator data we expected to find Siberian and Alaskan birds in the Eastern part of the wintering range [33], namely Kenya. Unexpectedly, however, Kenyan *oenanthe* were only slightly bigger than *oenanthe* from the rest of the wintering range, their wings not being longer and more pointed as we would have expected. Even more strikingly, their δ^2^H values were comparable to the other *oenanthe* birds, while we would have expected them to be much lower as indicated from isoscape maps [49]. However, there are other populations that are likely to spend the winter in the Eastern range, such as Arabian or Caucasian birds. We cannot exclude the possibility that the samples we obtained originated from such birds. Since we only collected samples at one single site, it is possible that populations are so distinctly segregated that birds found at one single site are all from the same or from close populations. With the due caution, this might be an indication of high connectivity between winter and summer populations in the Eastern populations of northern wheatear. The two additional geolocator studies tracked *oenanthe* birds from Sweden and Germany [34, 55]. These birds all spent the winter in the Western to central-Western Sahel, but the wintering area was rather broad, indicating relatively low connectivity. In this study we were able to differentiate birds found in Mauritania/Mali from birds found in Niger in 34 out of 40 cases. The fact that the assignments were not completely accurate might be an additional proof of the wide area used by different populations during the winter. However, the differing isotopic signatures in birds from Niger (males in particular) might indicate a higher concentration of central-Eastern European populations in this area, while birds wintering further West (where they also mix with Arctic *leucorhoa* birds and Western North African *seebohmi* birds) might originate from rather Western areas from Europe.

An additional interesting observation was the large sex bias we found in Niger, where we sampled 29 males and only 8 females. Personal observations confirmed that the bias was not related to trapping effort but to an actual overshoot of males in that area. Even though only anecdotal, this is an indication of sexual segregation in the wintering range, which might in part also explain the patterns of protandry observed in this species during spring migration (but see [78]).

This study shows that using a combination of different morphological and biochemical markers it is possible to differentiate birds in their wintering range with relatively high accuracy. Our data allow some speculation about the provenance of these birds even in the absence of reference materials from the breeding grounds. These considerations are rather speculative and unfortunately we lack indications from e.g. ringing recoveries to make clearer predictions. Reference material from the breeding grounds or additional tracking studies might shed lights on connectivity patterns in this area. Such material is crucial in order to correctly assign wintering birds to their breeding range. We suggest that a collection of the largest number of markers possible is the best way to refine assignments of this kind.

## Additional file

Additional file 1: Wheatears Africa. (CSV 12 kb)

## References

[1] Peach W, Baillie S, Underhill L (1991). Survival of British Sedge Warblers *Acrocephalus schoenobaenus* in relation to west African rainfall. Ibis.

[2] Ebbinge BS, Spaans B (1995). The importance of body reserves accumulated in spring staging areas in the temperate zone for breeding in dark-bellied brent geese *Branta bernicla bernicla* in the high arctic. J Avian Biol.

[3] Bairlein F, Henneberg H (2000). Der Weißstorch (*Ciconia ciconia*) im Oldenburger Land.

[4] Baker AJ, Gonzalez PM, Piersma T, Niles LJ, Nascimento IL S d, Atkinson PW (2004). Rapid population decline in red knots: fitness consequences of decreased refuelling rates and late arrival in Delaware Bay. Proc R Soc Lond B.

[5] Bearhop S, Hilton GM, Votier SC, Waldron S (2004). Stable isotope ratios indicate that body condition in migrating passerines is influenced by winter habitat. Proc R Soc Lond B.

[6] Cook JG, Johnson BK, Cook RC, Riggs RA, Delcurto T, Bryant LD, Irwin LL (2004). Effects of summer-autumn nutrition and parturition date on reproduction and survival of elk. Wildl Monogr.

[7] Norris DR, Marra PP, Kyser TK, Sherry T, Ratcliffe LM (2004). Tropical winter habitat limits reproductive success on the temperate breeding grounds in a migratory bird. Proc R Soc Lond B.

[8] Gunnarsson TG, Gill JA, Newton J, Potts PM, Sutherland WJ (2005). Seasonal matching of habitat quality and fitness in a migratory bird. Proc R Soc Lond B.

[9] Harrison XA, Blount JD, Inger R, Norris DR, Bearhop S (2011). Carry-over effects as drivers of fitness differences in animals. J Anim Ecol.

[10] Vickery JA, Ewing SR, Smith KW, Pain DJ, Bairlein F, Škorpilová J, Gregory RD (2014). The decline of Afro-Palaearctic migrants and an assessment of potential causes. Ibis.

[11] Marra PP, Hobson KA, Holmes RT (1998). Linking winter and summer events in a migratory bird by using stable-carbon isotopes. Science.

[12] Gill JA, Norris K, Potts PM, Gunnarsson TG, Atkinson PW, Sutherland WJ (2001). The buffer effect and large-scale population regulation in migratory birds. Nature.

[13] Bearhop S, Fiedler W, Furness RW, Votier SC, Waldron S, Newton J (2005). Assortative mating as a mechanism for rapid evolution of a migratory divide. Science.

[14] Studds CE, Marra PP (2005). Nonbreeding habitat occupancy and population processes: an upgrade experiment with a migratory bird. Ecology.

[15] Gunnarsson TG, Gill JA, Atkinson PW, Gelinaud G, Potts PM, Croger RE (2006). Population-scale drivers of individual arrival times in migratory birds. J Anim Ecol.

[16] Buehler DM, Piersma T (2008). Travelling on a budget: predictions and ecological evidence for bottlenecks in the annual cycle of long-distance migrants. Phil Trans R Soc B.

[17] Leyrer J, Lok T, Brugge M, Spaans B, Sandercock BK, Piersma T (2013). Mortality within the annual cycle: seasonal survival patterns in Afro-Siberian Red Knots *Calidris canutus canutus*. J Ornithol.

[18] Lozano GA, Perreault S, Lemon RE (1996). Age, arrival date and reproductive success of male American Redstarts *Setophaga ruticilla*. J Avian Biol.

[19] Sandberg R, Moore FR (1996). Fat stores and arrival on the breeding grounds: reproductive consequences for passerine migrants. Oikos.

[20] Kokko H (1999). Competition for early arrival in migratory birds. J Anim Ecol.

[21] Currie D, Thompson DBA, Burke T (2000). Patterns of territory settlement and consequences for breeding success in the northern wheatear *Oenanthe oenanthe*. Ibis.

[22] Bêty J, Gauthier G, Giroux J-F (2003). Body condition, migration, and timing of reproduction in snow geese: a test of the condition-dependent model of optimal clutch size. Am Nat.

[23] Smith RJ, Moore FR (2005). Arrival timing and seasonal reproductive performance in a long-distance migratory bird. Behav Ecol Sociobiol.

[24] Reudink MW, Marra PP, Kyser TK, Boag PT, Langin KM, Ratcliffe LM (2009). Non-breeding season events influence sexual selection in a long-distance migratory bird. Proc R Soc Lond B.

[25] Cooper NW, Murphy MT, Redmond LJ, Dolan AC (2011). Reproductive correlates of spring arrival date in the Eastern Kingbird *Tyrannus tyrannus*. J Ornithol.

[26] Descamps S, Bêty J, Love OP, Gilchrist HG (2011). Individual optimization of reproduction in a long-lived migratory bird: a test of the condition-dependent model of laying date and clutch size. Funct Ecol.

[27] Gordo O, Tryjanowski P, Kosicki JZ, Fulín M (2013). Complex phonological changes and their consequences in the breeding success of a migratory bird, the white stork *Ciconia ciconia*. J Anim Ecol.

[28] Rockwell SM, Bocetti CI, Marra PP (2013). Carry-over effects of winter climate on spring arrival date and reproductive success in an endangered migratory bird, Kirtland’s warbler (*Setophaga kirtlandii*). Auk.

[29] Jouventin P, Weimerskirch H (1990). Satellite tracking of wandering albatrosses. Nature.

[30] Trierweiler C, Klaassen RGH, Drent RH, Exo K-M, Komdeur J, Bairlein F, Koks BJ (2014). Population specific migration routes and migratory connectivity in a long-distance migratory raptor. Proc R Soc Lond B.

[31] Willemoes M, Strandberg R, Klaassen RH, Tψttrup AP, Vardanis Y, Howey PW (2014). Narrow-front loop migration in a population of the common cuckoo *Cuculus canorus*, as revealed by satellite telemetry. PLoS One.

[32] Bächler E, Hahn S, Schaub M, Arlettaz R, Jenni L, Fox JW (2010). Year-round tracking of small trans-Saharan migrants using light-level geolocators. PLoS One.

[33] Bairlein F, Norris DR, Nagel R, Bulte M, Voigt CC, Fox JW (2012). Cross-hemisphere migration of a 25 g songbird. Biol Lett.

[34] Schmaljohann H, Buchmann M, Fox JW, Bairlein F (2012). Tracking migration routes and the annual cycle of a trans-Sahara songbird migrant. Behav Ecol Sociobiol.

[35] Tøttrup AP, Klaassen RHG, Strandberg R, Thorup K, Kristensen MW, Jørgensen PS (2012). The annual cycle of a trans-equatorial Eurasian-African passerine migrant: different spatio-temporal strategies for autumn and spring migration. Proc R Soc Lond B.

[36] Hill GE (1993). Geographic variation in the carotenoid plumage pigmentation of male house finches (*Carpodacus mexicanus*). Biol J Linn Soc.

[37] Blackburn TM, Gaston KJ, Loder N (1999). Geographic gradients in body size: a clarification of Bergmann’s rule. Div Distrib.

[38] Lehtonen PK, Laaksonen T, Artemyev AV, Belskii E, Both C, Bureš S (2009). Geographic patterns of genetic differentiation and plumage colour variation are different in the pied flycatcher (*Ficedula hypoleuca*). Mol Ecol.

[39] Chandler CR, Mulvihill RS (1990). Wing-shape variation and differential timing of migration in dark-eyed juncos. Condor.

[40] Marchetti K, Price T, Richman A (1995). Correlates of wing morphology with foraging behaviour and migration distance in the genus *Phylloscopus*. J Avian Biol.

[41] Lockwood R, Swaddle JP, Rayner JMV (1998). Avian wingtip shape reconsidered: wingtip shape indices and morphological adaptations to migration. J Avian Biol.

[42] Fiedler W (2005). Ecomorphology of the external flight apparatus of blackcaps (*Sylvia atricapilla*) with different migration behavior. Ann NY Acad Sci.

[43] Förschler MI, Bairlein F (2011). Morphological shifts of the external flight apparatus across the range of a passerine (Northern Wheatear) with diverging migratory behaviour. PLoS One.

[44] Chamberlain CP, Blum JD, Holmes RT, Feng X, Sherry TW, Graves GR (1996). The use of isotope tracers for identifying populations of migratory birds. Oecologia.

[45] Hobson KA, Wassenaar LI (1996). Linking breeding and wintering grounds of neotropical migrant songbirds using stable hydrogen isotopic analysis of feathers. Oecologia.

[46] Hobson KA (1999). Tracing origins and migration of wildlife using stable isotopes: a review. Oecologia.

[47] Rubenstein DR, Chamberlain CP, Holmes RT, Ayres MP, Waldbauer JR, Graves GR, Tuross NC (2002). Linking breeding and wintering ranges of a migratory songbird using stable isotopes. Science.

[48] Hobson KA, Bowen GJ, Wassenaar LI, Ferrand Y, Lormee H (2004). Using stable hydrogen and oxygen isotope measurements of feathers to infer geographical origins of migrating European birds. Oecologia.

[49] Bowen GJ, Wassenaar LI, Hobson KA (2005). Global application of stable hydrogen and oxygen isotopes to wildlife forensics. Oecologia.

[50] Hobson KA, Waasenaar LI (2008). Tracking animal migration with stable isotopes.

[51] Collar N, de Juana E, del Hoyo J, Elliott A, Sargatal J, Christie DA, de Juana E (2013). Northern Wheatear (*Oenanthe oenanthe*). Handbook of the Birds of the World Alive.

[52] International BL (2004). Birds in Europe II: Population Estimates, Trends and Conservation Status.

[53] Maggini I, Bairlein F (2010). Endogenous rhythms of seasonal migratory body mass changes and nocturnal restlessness in different populations of northern wheatear *Oenanthe oenanthe*. J Biol Rhythms.

[54] Maggini I, Bairlein F (2012). Innate sex differences in the timing of spring migration in a songbird. Plos One.

[55] Arlt D, Olsson P, Fox JW, Low M, Pärt T (2015). Prolonged stopover duration characterizes migration strategy and constraints of a long-distance migrant songbird. Anim Migr.

[56] Delingat J, Hobson KA, Dierschke V, Schmaljohann H, Bairlein F (2011). Morphometrics and stable isotopes differentiate populations of northen wheatears (*Oenanthe oenanthe*). J Ornithol.

[57] Maggini I, Spina F, Voigt CC, Ferri A, Bairlein F (2013). Differential migration and body condition in northern wheatears (*Oenanthe oenanthe*) at a Mediterranean stopover site. J Ornithol.

[58] Jenni L, Winkler R (1994). Moult and ageing of European passerines.

[59] Svensson L (1992). Identification guide to European passerines.

[60] Bairlein F (1995). European-African songbird migration network: Manual of Field Methods, revised edition.

[61] Wassenaar LI (2008). An introduction to light stable isotopes for use in terrestrial animal migration studies. Terr Ecol.

[62] Erzberger A, Popa-Lissenau A, Lehmann GUC, Voigt CC (2011). Potential and limits in detecting altitudinal movements of bats using stable hydrogen isotope ratios of fur keratin. Acta Chiropterol.

[63] Wassenaar LI, Hobson KA (2003). Comparative equilibration and online technique for determination of non-exchangeable hydrogen of keratins for use in animal migration studies. Isot Environ Health Stud.

[64] Wassenaar LI, Hobson KA (2000). Improved method for determining the stable-hydrogen isotopic composition (δD) of complex organic materials of environmental interest. Environ Sci Technol.

[65] R Core Team (2013). R: A language and environment for statistical computing.

[66] Browne PWP (1982). Palaearctic birds wintering in southwest Mauritania: species, distribution and population estimates. Malimbus.

[67] Förschler MI, Metzger B, Maggini I, Neumann R, Bairlein F (2009). Seebohm’s wheatear *Oenanthe oenanthe seebohmi* in West Africa. Bull Afr Bird Club.

[68] Meehan TD, Giermakowski JT, Cryan PM (2004). GIS-based model of stable hydrogen isotope ratios in North American growing-season precipitation for use in animal movement studies. Isot Environ Health St.

[69] Smith BN, Epstein S (1971). Two categories of ^13^C/^12^C ratios for higher plants. Plant Physiol.

[70] Lajtha K, Marshall JD, Lajtha K, Michener RH (1994). Sources of variation in the stable isotopic composition of plants. Stable isotopes in ecology and environmental sciences.

[71] Kelly JF (2000). Stable isotopes of carbon and nitrogen in the study of avian and mammalian trophic ecology. Can J Zool.

[72] Heaton THE (1987). The ^15^N/^14^N ratios of plants in South Africa and Namibia: relationship to climate and coastal/saline environments. Oecologia.

[73] Hobson KA, Alisauskas RT, Clark RG (1993). Stable-nitrogen isotope enrichment in avian tissues due to fasting and nutritional stress: implications for isotopic analysis of diet. Condor.

[74] Hobson KA (1999). Stable-carbon and nitrogen isotope ratios of songbird feathers grown in two terrestrial biomes: implications for evaluating trophic relationships and breeding origins. Condor.

[75] Hebert CE, Wassenaar LI (2001). Stable nitrogen isotopes in waterfowl feathers reflect agricultural land use in western Canada. Environ Sci Technol.

[76] Hartman G, Danin A (2010). Isotopic values of plants in relation to water availability in the Eastern Mediterranean region. Oecologia.

[77] Pyankov VI, Ziegler H, Akhani H, Deigele C, Lüttge U (2010). European plants with C_4_ photosynthesis: geographical and taxonomic distribution and relations to climate parameters. Bot J Linn Soc.

[78] Schmaljohann H, Meier C, Arlt D, Bairlein F, van Oosten H, Morbey YE (2016). Proximate causes of avian protandry differ between subspecies with contrasting migration challenges. Behav Ecol.

